# Cholangioscopic evaluation of distal bile duct stones and intraductal lesions in a patient with anomalous junction of pancreaticobiliary ducts and pancreatic duct anomaly

**DOI:** 10.1055/a-2740-3992

**Published:** 2025-12-08

**Authors:** Shiyu Zhang, Xun Zhang, Yi Tu, Xiaojiang Zhou, Raymond Shing Yan Tang, Junbo Hong

**Affiliations:** 1 117970 Department of Gastroenterology, The First Affiliated Hospital of Nanchang University, Nanchang, China; 2 117970 Department of Pathology, The First Affiliated Hospital of Nanchang University, Nanchang, China; 3 13621 Department of Medicine and Therapeutics, Prince of Wales Hospital, Hong Kong, China

Cholangioscopy is now widely performed in patients with pancreaticobiliary disorders, including difficult common bile duct (CBD) stones and indeterminate biliary strictures. Furthermore, an increasing number of rare pancreaticobiliary disorders have been identified by cholangioscopy when fluoroscopy fails to provide a definitive diagnosis 1 2 3 4 .

A 46-year-old woman was admitted for the treatment of a distal CBD stone, which cannot be identified under percutaneous cholangioscopy through the T-tube after laparoscopy cholecystectomy and CBD exploration. Fluoroscopy suggested an irregular stone in the distal CBD and choledochal cyst. Due to the unavailability of laser or electrohydraulic lithotripsy devices, the stone could not be captured with the retrieval basket under cholangioscopic guidance. Finally, following unsuccessful attempts with foreign body forceps, the CBD stone was successfully extracted using a standard retrieval basket under fluoroscopic guidance ( Fig. 1 **a–c** ).

**Fig. 1:**
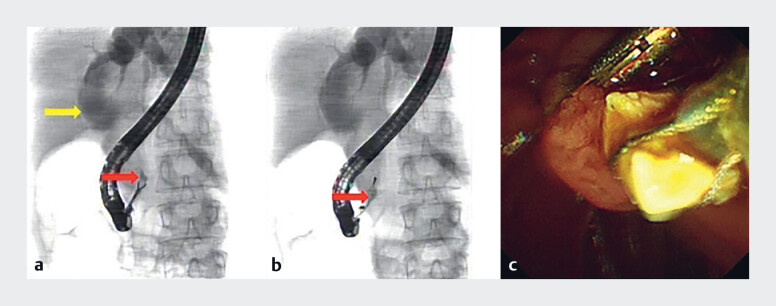
Fluoroscopy and duodenoscopy. **a** Fluoroscopy revealed an irregular stone in the distal CBD and choledochal cyst, and an unsuccessful stone extraction attempt using foreign body forceps (red arrow: distal CBD stone and yellow arrow: choledochal cyst). **b** The CBD stone was successfully extracted using a standard retrieval basket under fluoroscopic guidance (red arrow). **c** A duodenoscope revealed the CBD stone.

While CBD stone clearance was confirmed on cholangioscopy, an intraductal protruding lesion with intensely hyperemic and irregular surface was observed near the biliary orifice. Additionally, multiple openings were noted near the biliary orifice, which were subsequently shown to be pancreatic duct orifices upon fluoroscopic-guided guidewire cannulation ( Fig. 2 **a–d** ). Cholangioscopy-guided biopsy of the lesion revealed nonspecific inflammation on histology ( Video 1 and Fig. 3 ).

**Fig. 2:**
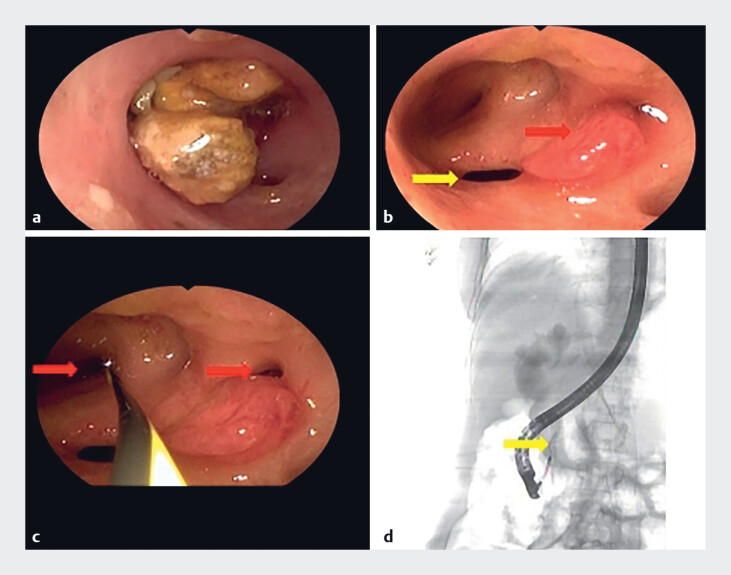
Cholangioscopy and fluoroscopy. **a** A CBD stone was identified under the cholangioscopy. **b** A protruding lesion with an intensely hyperemic and irregular surface was observed near the biliary orifice (red arrow: intraductal neoplasm and yellow arrow: common bile duct orifice). **c, d** Multiple openings were noted under cholangioscopy, and fluoroscopic-guided guidewire cannulation demonstrated that these represented pancreatic duct orifices (red arrow: pancreatic duct orifices and yellow arrow: pancreatic duct).

Cholangioscopy-guided biopsy.Video 1

**Fig. 3:**
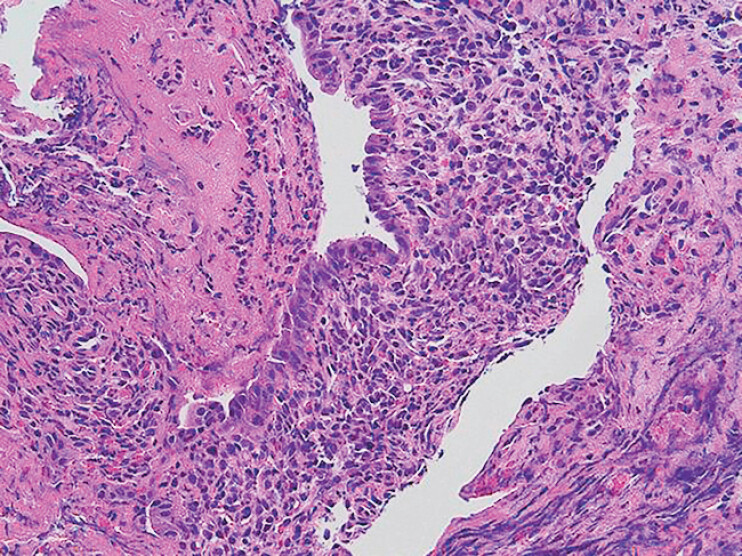
Histology for intraductal neoplasm. The biliary epithelium exhibits a preserved architecture with a dense mixed acute and chronic inflammatory infiltrate.

Follow-up cholangioscopy after 9 months documented the complete resolution of the previously identified lesion near the biliary orifice, with no abnormal findings within the choledochal cyst ( Fig. 4 ).

**Fig. 4:**
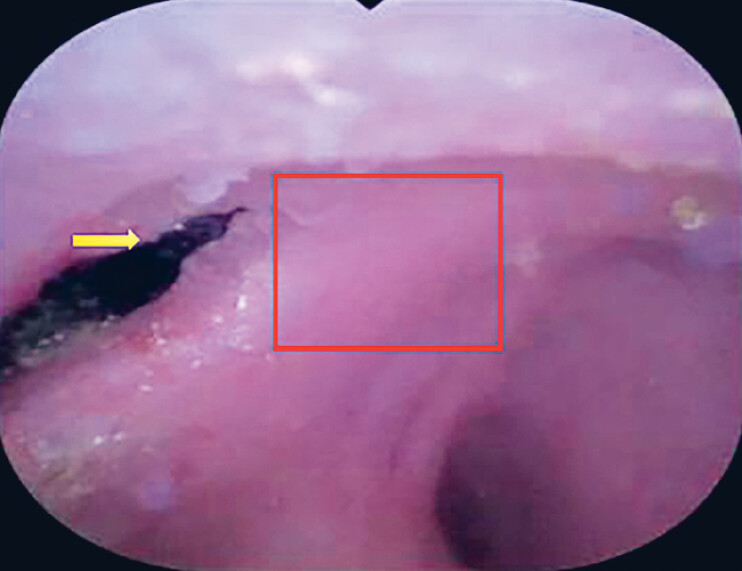
Complete resolution of the previously identified lesion (red frame). Yellow arrow: common bile duct orifice.

In this patient, the anomalous junction of pancreaticobiliary ducts with concomitant pancreatic duct anomaly and choledochal cyst can be directly observed on cholangioscopy. These pathological changes resulted in choledocholithiasis and an intraductal inflammatory mass that regressed spontaneously after CBD stone removal. Given the malignant transformation potential of choledochal cysts 5 , surgical resection was advised, but the patient declined the procedure.

Endoscopy_UCTN_Code_TTT_1AR_2AB

## Notes

Endoscopy E-Videos https://eref.thieme.de/e-videos
*E-Videos* is an open access online section of the journal *Endoscopy* , reporting on interesting cases and new techniques in gastroenterological endoscopy. All papers include a high-quality video and are published with a Creative Commons CC-BY license. Endoscopy E-Videos qualify for HINARI discounts and waivers and eligibility is automatically checked during the submission process. We grant 100% waivers to articles whose corresponding authors are based in Group A countries and 50% waivers to those who are based in Group B countries as classified by Research4Life (see: https://www.research4life.org/access/eligibility/ ). This section has its own submission website at https://mc.manuscriptcentral.com/e-videos .
